# Does HLA matching matter in the modern era of renal transplantation?

**DOI:** 10.1007/s00467-019-04393-6

**Published:** 2019-12-09

**Authors:** Jon Jin Kim, Susan V Fuggle, Stephen D Marks

**Affiliations:** 1 Department of Paediatric Nephrology, Nottingham Children’s Hospital, Nottingham, NG7 2UH UK; 2 Institute of Medical Sciences, Faculty of Medicine, University of Liverpool, Liverpool, L69 3BX UK; 3 NHS Blood and Transplant, Bristol, BS34 8RR UK; 4 Oxford Transplant Centre, Nuffield Department of Surgical Sciences, University of Oxford, Oxford, OX3 7LE UK; 5 Department of Paediatric Nephrology, Great Ormond Street Hospital for Children NHS Foundation Trust, London, WC1N 3JH UK; 6 NIHR Great Ormond Street Hospital Biomedical Research Centre, University College London Great Ormond Street Institute of Child Health, London, WC1N 1EH UK

**Keywords:** Human leukocyte antigens, Matching, Mismatching, Living donors, Deceased donors, Kidney transplantation

## Abstract

Children with end-stage kidney disease should be offered the best chance for future survival which ideally would be a well-matched pre-emptive kidney transplant. Paediatric and adult practice varies around the world depending on geography, transplant allocation schemes and different emphases on living (versus deceased) donor renal transplantation. Internationally, paediatric patients often have priority in allocation schemes and younger donors are preferentially allocated to paediatric recipients. HLA matching can be difficult and may result in longer waiting times. Additionally, with improved surgical techniques and modern immunosuppressive regimens, how important is the contribution of HLA matching to graft longevity? In this review, we discuss the relative importance of HLA matching compared with donor quality; and long-term patient outcomes including re-transplantation rates. We share empirical evidence that will be useful for clinicians and families to make decisions about best donor options. We discuss why living donation still provides the best allograft survival outcomes and what to do in the scenario of a highly mismatched living donor.

## Introduction

The first successful kidney transplant was performed in 1954 and was possible because of the perfect genetic match between the Herrick brothers who are identical twins [1]. At around the same time, the proteins that differentiate self from non-self were identified and called the human leukocyte antigens (HLA) [2]. Detection of antibodies against donor HLA using the cell-dependent cytotoxicity (CDC) crossmatch, published by Terasaki in 1969, reduced the rates of hyperacute rejection and made transplantation safer [3]. The subsequent development of immunosuppression, initially with corticosteroids and azathioprine, enabled transplantation to be a long-term therapeutic viability. However, immunosuppression has become more effective (with the introduction of ciclosporin, tacrolimus and mycophenolate mofetil in the 1990s and subsequently with induction agents, such as basiliximab). This together with improved surgical techniques has resulted in improved patient and renal allograft survival outcomes [4]. Currently, the expected 5-year survivals of paediatric living and deceased donor kidney transplants are > 85% and > 75% respectively [5, 6]. We therefore question whether HLA matching is still relevant in the modern transplant era.

The major histocompatibility complex (MHC) is located on a large 4Mbp portion of chomosome 6p21 and encodes the HLA proteins [7]. HLA are essential for differentiation of self from non-self and the extensive polymorphism of HLA has been maintained throughout evolution. The conventional classification divides HLA into class I and class II. HLA class I (HLA-A, B and C) are expressed on nearly all nucleated cells and load anomalous peptides (e.g. viral peptides) on MHC class I and signal to CD8 T cells for killing. HLA class II (HLA-DR, DQ and DP) are located on professional antigen-presenting cells (APC), in particular dendritic cells and B cells, which can sense antigens, engulf, process and load the antigens to activate CD4 T cells. Therefore, the genetic polymorphism is important to ensure presentation of a wide repertoire of foreign antigens. This in turn presents a significant barrier to transplantation and underlies the process of rejection. Of note, the name HLA is a misnomer as they serve an important natural role and as such are not antigens for the body’s immune system.

## Lessons from registries

Human leukocyte antigens matching has been at the forefront of transplantation so there are no randomised controlled trials studying its role. Geography is important to consider in this context as cold ischaemia times would be increased in large countries if deceased donor organs were travelling huge distances, whereas HLA matching is prioritised in the UK, which is a relatively small country. These practical considerations and the relative size of deceased donor programs are reflected in the different practices of organ allocation policies around the world [8]. Having a larger deceased donor pool would increase flexibility in donor allocation criteria. In certain countries however, deceased donor programs have not been set-up and the only option would therefore be living donation.

Paediatric patients who have pre-emptive transplantation unequivocally have better patient and renal allograft survival compared with dialysis-exposed patients [9]. This effects both living and deceased donation; and survival decreases as dialysis times go over 1 year. Patients who are pre-emptively transplanted have different demographics though, with a higher proportion of structural defects, higher proportion of white non-Hispanics and lower social deprivation. Nonetheless, the registry data support policies to improve earlier detection of kidney disease and streamlined pathways to access transplantation.

The evidence behind HLA matching derives from macro-level population studies from national and multi-national registries. There are caveats from interpreting registry results including quality of registry data (e.g. data completeness, accuracy and co-variates) and methodology for data analysis (e.g. pre-specified hypotheses and co-variates, statistical analysis and comparator groups) [10]. Data from some registries do not include all clinical events over time such as rejection or changes in treatment. Most national registries mandate data collection although it is difficult to compare the results due to different patient populations, healthcare systems and data collection methods (see Table 1) [11, 12].

**Table 1:** Summary of main papers from registries around the world. This is not meant as a critique but highlights the different approaches and results from each paper

Author, registry	Year, number of patients	Research question	Co-variates											Main results	Ref
			A	B	C	D	E	F	G	H	I	J	K		
Gritsch (2008), OPTN	1996–2004, 2292 DD < 18 years	HLA-DR MM and donor age	1	1	√	√	√	√	√	√	√			Allograft survival at 5 years was similar for each HLA-DR MM. Donors > 35 years of age had worse survival.	31
Opelz (2010), CTS	1998–2007, 9209 < 18 years	HLA MM and donor age	1	1	√		√	√	√	√	√	√		Survival rates were similar for donors up until the age of 49. HLA MM increases risk of graft failure in a hierarchal manner and this effect persisted with modern immunosuppression. PTLD is associated with two HLA-DR MM.	16
Foster (2013), USRD	1994–2004, FU 2009 9358 < 21 years	Relative importance of HLA MM and donor age	1	1	√	√	√	√	√	√	√		√	HLA MM and donor age associated with DD survival. Advantages of younger donors (< 35 years) offset poor HLA MM. Donors > 45 years with 0–1 MM had similar survival to younger donors. Older LD with poor HLA MM had similar outcomes to DD.	14
Marlais (2016), UKTR	2000–2011, 1378 < 18 years	HLA MM	1	√	√	√	√	√	√	√	√			Poorly matched LD are not inferior when compared with well-matched DD.	18
Opelz (2017), CTS	2000–2015, 3627 < 18 years	HLA MM	1	√	√		√	√	√	√	√	√		Graft survival is significantly associated with HLA MM both for individual HLA MM loci and ‘tier’ system. 4–6 HLA MM LD graft survival was worse compared with 0–1 HLA MM DD.	17
Chesneye (2017), ERA-EDTA	1990–2013, 4686 < 20 years	Donor and recipient age		1	√	√		√			√			DD age was non-linearly associated with graft survival. Highest risk of graft failure was donors < 5 years. Graft survival was similar for older LD compared with younger LD.	30
Williams (2018), USRD	1987–2016, 18,602 < 18 years	HLA MM	1	√	√	√		√		√		√		Kidney allograft survival is incrementally associated with HLA MM for DD and LD. 0–3 MM DD survival is equivalent to 4–6 MM LD.	15
Trnka (2018), ANZDATA	1990–2015, 1134 < 20 years	HLA MM and donor/recipient age difference	1	1	√	√	√	√		√	√	√		Graft loss of DD was consistently higher than LD at all time points. Both age difference and HLA MM were incrementally associated with graft loss.	19

*A*, donor age; *B*, HLA MM; *C*, recipient demographics including age, sex and race; *D*, primary renal disease; *E*, donor demographics including race 1, variable that is addressed as question in the study (excluding age); *F*, transplant era or year; *G*, cold ischaemic time; *H*, panel reactive antibodies; *I*, duration of dialysis or pre-emptive transplantation; *J*, immunosuppression; *K*, socioeconomic states

**Fig. 1:**
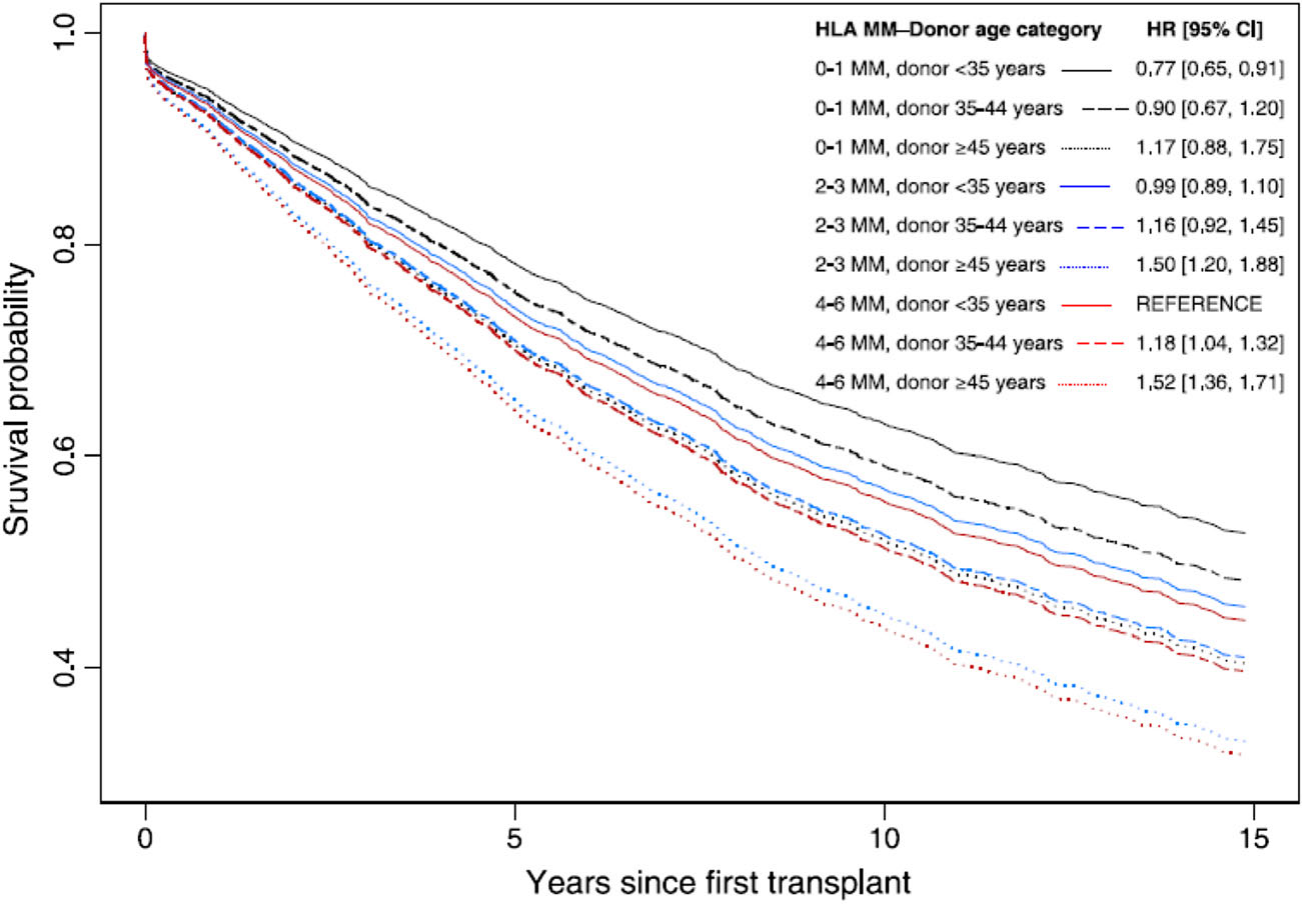
Comparison of donor age and HLA MM. Analysis was performed by Foster et al. using USRD [14]. Each curve shows the adjusted estimated graft survival for a white 14-year-old recipient transplanted in 1999–2004 from a DD with a different age and HLA MM combination. Also shown are the adjusted HRs from a model comparing graft survival for each combination compared to the reference 4–6 HLA MM donor of < 35 years of age. For donors < 35 years, 0–1 HLA MM had statistically significant improved allograft survival. Donors > 45 years had worse allograft survival though in the well-matched (0–1 MM) group, graft survival outcomes were similar. Therefore, both allograft quality and HLA matching are important for determining survival outcomes though there is a larger effect of donor quality in the earlier post-transplant years (5–10 years)

The United Network for Organ Sharing (UNOS) registry in the USA contains one of the largest and oldest sets of patients. An early report looking at the period from 1987 to 1998 (8422 patients under 21 years) showed a large centre effect without statistically significant difference in renal allograft survival due to HLA mismatch (MM) [13]. A later cohort from 1994 to 2004 (9358 patients under 21 years) in the modern immunosuppression era was investigated for the effects of HLA matching and donor age [14]. Overall, the risk of allograft loss increased with higher MM for both deceased donor (DD) and living donor (LD) transplants. Older donors classified as ≥ 45 years were associated with worse outcomes in DD but there was no association found in LD (Fig. 1). This study was unique as estimated socioeconomic status was included as a variable but did not reach statistical significance in the final model. Williams et al. recently analysed the entire cohort from 1987 to 2016 with a total of 18,602 patients under 18 years of age [15]. There was a strong effect of transplant era and recipient age. Recipients in the oldest fourth quartile (from 15 to 18 years) had a hazard ratio (HR) of 1.76 compared with the lowest quartile (0 to 7 years) and the HR increased with each age quartile. The fully adjusted co-variate model also included donor details, underlying diagnosis and discharge immunosuppression. Risk of allograft loss increased with each HLA MM in a linear order for both LD and DD. There was a 16% higher risk of allograft loss for 1 MM in DD and 62% for 5 and 6 MM. The size effect of MM was higher in LD with 48% for 1 MM and 114% for 6 MM though overall survival rates of LD transplants were as long or longer than DD. The number of LD with 4 to 6 MM was smaller, as expected, as the majority of LD were haplotype matched parents.

Similar results are obtained when examining other worldwide registries. The Collaborative Transplant Study (CTS) is a worldwide co-operative effort with input from 42 countries. Opelz and Dӧhler analysed the cohort from 1988 to 2007 which included 9209 patients under 18 years of age [16]. The analysis was stratified into two decades, 1988 to 1997 and 1998 to 2007, with consistently better outcomes in the latter era. Patients with 4 to 6 MM had an HR of allograft loss of 1.19 (95% confidence interval, CI 1.08–1.32) compared with 0 to 3 MM in the early decade and this was similar in the second decade (HR 1.26, *p* = 0.009) despite better immunosuppression. A more recent analysis was performed for patients from 2000 to 2015 (3627 LD only transplants) [17]. Compared with 0 MM LD (most likely fully matched siblings), the group of 1 to 3 MM had an HR of 2.2 (CI 1.39–3.49) and 4 to 6 MM had an HR of 3.91 (CI 2.37–6.45). Therefore, the effect of HLA MM in LD was clustered into two groups, 1 to 3 MM and 4 to 6 MM, which follows donor-recipient relationship categories, i.e. haplotype matched first-degree relatives (mostly parents) and non-haplotype matched donors (living related or unrelated). Haplotype matched donors will also have benefits of matching additional minor histocompatibility antigens (non-HLA polymorphisms able to elicit an immune response). The HR of HLA MM in this study is high as the reference group was the full 0 MM LD. Fully matched siblings represent the closest to a perfect match which would be identical twins. In the UK Transplant Registry cohort of 1378 patients from 2000 to 2011, worse allograft outcomes were associated with poorer HLA matching with HLA matching performed using a tier system [18]. In the Australia and New Zealand Dialysis and Transplant Registry (ANZDATA), adjusted HR increased 20% for each HLA MM in the 1134 patients analysed from 1990 to 2015 [19]. Therefore, as a whole, HLA MM significantly increases the risk of graft failure for both groups of LD and DD recipients. The risks are incremental for each HLA MM for DD. The risks tend to group according to the relationship to the donors for LD (haplotype matched parents/siblings or non-haplotype matched donors).

## Comparing HLA class I and class II

The evidence of an HLA loci effect is contentious with conflicting results comparing HLA-A versus HLA-B versus HLA-DR or HLA class I versus class II. Evidence for deleterious effects of HLA-DR MM came from older studies [20]. HLA class II had no statistically significant effect on 5-year outcomes in the modern immunosuppression era (post-1994) in the CTS report [16]. Williams et al. also found no effect when comparing all 27 permutations of HLA MM. The authors concluded that the effect of HLA MM is therefore additive and does not depend on particular HLA loci [15].

More recently, long-term allograft loss is often associated with antibody-mediated rejection (ABMR). ABMR is more prevalent amongst HLA class II antibodies, and in particular HLA-DQ [21, 22]. The target for HLA class II antibodies is being debated although biopsies show evidence of microcirculatory inflammation. It has been long known that vascular endothelial cells and renal tubules can upregulate MHC class II during inflammation [23–25]. Nocera et al. published an elegant study eluting HLA antibodies from kidney biopsies [26]. HLA antibodies in the graft were only detected in the presence of serum HLA antibodies and were of identical HLA specificities. Graft HLA antibodies were again predominantly HLA class II (76%), compared with 24% HLA class I. It is not clear if the eluted graft antibodies were produced by intra-graft plasma B cells or from circulating or lymphoid B cells.

If HLA class II MM drives ABMR, why is allograft outcome not worse with HLA class II MM in registry studies? This could be due to inherent methodological limitations that cannot take into account the interaction between HLA class I and class II antibodies. In addition, HLA-DQ MM is currently not consistently recorded. On many occasions when HLA-DR is matched, HLA-DQ also is matched because of linkage disequilibrium, but this is not always the case [27]. In addition, the DQ (and DP) molecules consist of two chains (α and β) both of which are polymorphic and can exist in *cis* and *trans* isomers [21]. Therefore, there are four possible combinations of HLA-DQ which potentially complicate incorporating HLA-DQ matching in kidney matching schemes. In adult registry studies, Lim et al. recently showed that HLA-DQ MM increased the likelihood of acute rejection and ABMR (all patients in ANZDATA) [27]. HLA-DQ MM alone was not a risk factor for allograft loss but the combination of HLA-DR and DQ MM had the worst outcome [27, 28]. Incorporating HLA-DQ MM information in future registry studies will further elucidate the importance of HLA class II versus class I matching.

## Kidney donor quality

Expanded criteria donors (ECD) are defined as donors over 60 years or over 50 years of age with at least two risk factors of hypertension, serum creatinine > 1.5 mg/dl or death by cerebrovascular accident. ECD carry a higher risk of allograft failure and are not commonly used for paediatric recipients [29]. The risk factor of donor age as a marker of kidney quality has been widely studied [14, 16, 19, 30]. The importance of donor age was exemplified by the ‘Share 35’ kidney allocation policy in the USA which was implemented in November 2005. ‘Share 35’ gave absolute priority for donors under 35 years to be allocated to paediatric recipients, regardless of HLA matching. This resulted in reduced time to transplantation for children on the deceased donor waiting list and impacted clinicians performing less living donor transplants which could also increase sensitisation in subsequent transplants. Gritsch et al. examined the data for 8 years prior to ‘Share 35’ and showed that donors over 35 years of age had a relative risk of allograft loss of 1.32 compared with donors under 35 years of age at 5-year follow-up and the effect was independent of HLA MM [31]. Therefore, they concluded that a good quality donor outweighs the benefit of HLA matching. A similar analysis was performed by Foster et al. but including patients up to 21 years of age [14]. To study the interaction between donor age and HLA MM, they created stratified groups based on a hypothetical patient. There was no effect of HLA MM on DD under 35 years of age but both HLA MM and donor age increased the risk of allograft failure with DD above 35 years of age. This was corroborated by the 2010 CTS analysis which also showed donors up to 49 years of age had equal outcomes [16]. Of note, follow-up analysis was limited to 5 years in these studies and the effects of donor age and HLA MM could diverge with longer follow-up.

The prognosis from DD under 10 years of age is worse due to increased risk of surgical complications including thrombosis, rejection and hyperfiltration injury [30]. The donor age effect is less marked in LD and allograft survival outcomes are comparable due to full evaluation of LD for fitness to donate. Outcomes of donation from grandparents are also comparable with parents [32]. There is therefore no strict age cutoff for LD providing there is careful donor assessment.

Does HLA MM matter more than quality? This cannot be answered by multivariate analyses as each factor is assessed with an average taken of all the other factors (proportional hazards assumption). When assessing HLA MM, the average donor age and other co-variates are calculated, and when assessing donor age, the average HLA MM is calculated. It does not compare HLA MM for each donor age category and vice versa. Therefore, the analysis of HLA MM and donor age can only be performed by creating categories of each combination as performed by Foster as mentioned above [14]. In addition, the effect of donor age is not linear as there is a ‘U-shaped’ effect of donor age in DD, with high risk for donors under 10 years of age and a gradual increase in risk with increasing donor age [30]. ERA-EDTA registry data showed gradual increase in the HR with donor age but the risk increased markedly after donors above 50 years of age. Studies that use donor age as a linear co-variate will potentially underestimate its effect.

Donor age alone does not fully capture information about donor quality. Donors are now assigned a donor score which is the kidney donor profile index (KDPI) in USA which consists of ten different factors [33]. HLA matching is accounted for by giving additional points for full HLA match (0 MM) and if not fully matched, points for HLA-DR matching. Early results are promising with improved 1-year allograft survival which are mainly improvements in the period early post-transplant [34].

## Living versus deceased donors

**Fig. 2:**
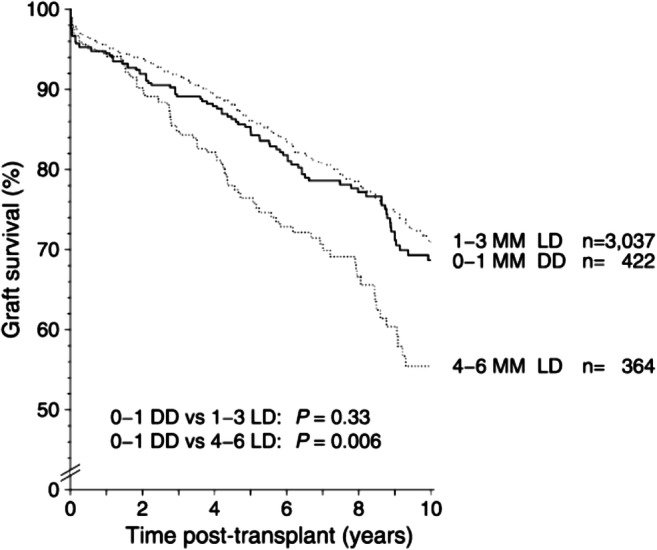
KM analysis of CTS data by Opelz et al. [17]. Poorly matched (4–6 MM) LD had worse allograft survival compared with a very well-matched (0–1 MM) DD. Likewise, transplants from a 1–3 MM LD had similar outcomes to the very well-matched (0–1 MM) DD group. These results and data from other studies support entering high HLA MM (4–6 MM) LD into kidney paired exchange schemes

Renal allograft survival is improved for recipients of LD compared with DD in most registry data. There are similar outcomes in general between older LD and DD [14]. Well-matched (0 to 3 MM) older LD have better allograft survival than poorly matched (4 to 6 MM) younger DD [14]. The majority of LD in paediatrics are from parents and first-degree relatives and are therefore haploid MM (0 to 3 MM). In addition, the quality of kidney is controlled as donors undergo strict donor assessment, albeit that higher risk LD (e.g. hypertensive donors) is now considered even for paediatric recipients. Cold ischaemia time and ischaemic reperfusion injury are significantly reduced resulting in markedly less delayed graft function. The effect of HLA MM therefore is unsurprisingly stronger in LD and there is a significant increase from the 1 to 3 MM than from the 4 to 6 MM. The question therefore arises, what is the best practice for 4 to 6 MM LD? In the most recent UNOS analysis, LD with 4 to 6 MM lost their LD advantage and had similar allograft survival for 0 to 3 MM DD [15]. Comparing with a more selective group of 0 to 1 MM DD, poorly matched 4 to 6 MM had worse allograft survival (HR 1.36, 1.09–1.69, *p* = 0.006) [17] (Fig. 2). Therefore, one must weigh the outcomes of a poorer match LD against the matchability or the probability of being successful in obtaining a better matched DD which may have additional implications for children on dialysis. This is potentially the case if grandparents or other extended relatives are being considered as LD. However, it is increasingly common in practice to put those donor-recipient pairs forward for the national living donor kidney sharing schemes.

## Long-term renal allograft survival

At present, the aim is for children to receive a renal transplant with good patient and allograft survival rates with the potential for pre-emptive re-transplantation in the future. There are paediatric renal transplant recipients who become adults with functioning renal allograft lasting more than 20 years [35–37]. However, there are no defining predictors for those long-term survivors, although these patients tended to have better HLA matching, younger donors and LD (as opposed to DD) donors. The effect of HLA matching becomes more pronounced over the years so for transplants that survive longer than 15 years, the Kaplan-Meier curves became more widely separated as demonstrated from UNOS data [15]. The median survival for 0 MM was 25 years compared with 20 years for 1 MM, 18 years for 2 MM, 15 years for 3 MM and 12 years for higher MM [15]. Allograft loss is primarily due to chronic immune injury and accounts for 50% of allograft losses followed by a distant second cause of disease recurrence (8%) [38]. There is still a significant risk of rejection even with modern immunosuppression, particularly ABMR which can present with a low-grade indolent course [22].

## Effects of HLA matching beyond the first transplant

Children with kidney transplants face a lifetime on immunosuppression. Morbidity and mortality are now mainly due to side effects of immunosuppression rather than renal failure itself [5, 38]. The cumulative incidence of cancers becomes significantly high after 25 years of transplant life; 27% for all cancers in the ANZDATA cohort and 22% in one Dutch study [39, 40]. In addition, 30% develop a second cancer within 2 years of the first cancer [40]. Squamous and basal cell skin cancers were the most predominant type followed by non-Hodgkin lymphoma (NHL)/post-transplant lymphoproliferative disorder (PTLD) and cervical cancers which may be preventable through HPV vaccination [41]. There was a bimodal distribution of cancer incidence, with a peak of 6.6 years for NHL/PTLD and 14.8 years for all others. The standardised incidence ratio for non-skin cancers was 8.23 (CI 6.9–9.7) compared with the healthy population and the risk of cancer was equivalent to a population 25 to 30 years older [39]. The risk of cancer increased with the length of time post-transplant without any significant differences between immunosuppression regimes [39, 40, 42]. The risk of PTLD was found to be associated with HLA-DR MM in one study but not corroborated by other studies [16, 39]. It would therefore be ideal to develop immunosuppression minimisation strategies based on HLA matching and clinical biomarkers.

Allograft failure also impacts waiting times for subsequent re-transplants [43, 44]. HLA sensitisation rates increased after allograft failure depending on the level of HLA MM [43]. Patients with 2 DR MM waited 23 months compared with 19 months for 0 to 1 HLA-DR MM patients [44]. In addition, a higher proportion of patients with 2 HLA-DR MM were still on the waiting list and had 32% less chance of being successfully transplanted. The HLA-DR MM of the first transplant also reduced survival in the second transplant and this result has been corroborated in a number of adult studies [7, 44]. Importantly though, HLA-DR MM due to a LD at the first transplant had no negative outcomes in the second transplant [44].

Foster et al. looked at retransplant rates in UNOS patients under 21 years of age between 1988 and 2009 [45]. HLA matchability was calculated so patients were not penalised twice for getting poor MM transplants because of difficulty matching the first transplant and also subsequent transplants. Socioeconomic status was also included in the model. In the first transplant, recipients with 4 to 6 HLA MM did worse compared with 0 to 1 and 2 to 3 MM but the effect only became more apparent 10 years post-transplant. This was significant for both HLA class I and class II MM. Increasing MM in the first transplant increased waiting time for the second transplant. The median waiting time for a previous 0 to 1 MM was 2 years compared with 3 years for 2 to 3 MM and 5 years for 4 to 6 MM [45].

## Future HLA matching strategies

Historically, HLA typing was performed using serological methods. HLA antibodies were identified in sensitised individuals (e.g. multiparous women and these antisera were used as HLA typing reagents). HLA antibodies were therefore clinically significant but the number of HLA types was limited by the available antibodies. HLA typing is now performed using molecular methods but high-resolution typing is not routinely used in typing donors and recipients for solid organ transplantation. Furthermore, all HLA specificities and loci are not individually considered in determining the level of an HLA match. Consequently a zero mismatch might not be fully matched. This can partly explain some of the benefits of LD versus DD. In a LD from direct family members, one can be certain of the HLA matches and the maximum actual mismatch is a haplotype 50% mismatch.

Computational three-dimensional reconstructions of HLA molecules can be derrived from the molecular types, although high-resolution HLA typing is required. Amino acids might be contiguous on the HLA molecule but adjacent to different amino acids in three-dimensional space. The eplet analysis through HLAMatchmaker takes this spacial configuration into account in defining three amino acid sequences as epitopes on the surface of the HLA molecule [46, 47]. HLA MM is performed based on a score of potential epitope mismatches between donor and recipient rather than limited to 0/1 for each allele. The epitope score for HLA-DR was shown to be a better predictor of the development of HLA-DR antibodies [48, 49]. Some patients with 0 HLA-DR MM had high eplet MM and vice versa. The tertiary HLA structure can also be computed based on the electrostatic interaction between amino acid molecules (EMS-3D score) [47, 50, 51]. The electrostatic forces are also postulated to affect affinity binding of antibody to HLA epitopes. Increasing EMS-3D was strongly and independently associated with an incremental increase in the risk of allograft failure in the adult cohort of 10,726 first DD patients in the UK Transplant Registry [52]. HLA matching will become more refined in the future, which would potentially change how organs are allocated. Matching would no longer be a dichotomous result but be based on a score. There would then be a threshold risk (e.g. 11/41 in the Wiebe et al. analysis) for the development of HLA antibodies or rejection [48].

## Conclusion

While the incidence of end-stage kidney disease (ESKD) is increasing in adults, the number of new paediatric patients in developed countries has plateaued [53]. Children represent only a small proportion of patients in renal transplantation programs. There needs to be consideration of the risks of sensitisation and re-transplantation in designing new kidney offering schemes for potential future paediatric renal transplant recipients. Kidney donor quality is fundamentally important, particularly for DD and also for LD. The medium- to long-term allograft survival for DD is highly influenced by donor quality. Allografts with very long-term survival though (> 15 years) are associated with better HLA matching. The main cause of allograft failure is still chronic immune injury despite advances in immunosuppression. Even patients with 0 HLA-A, HLA-B and HLA-DR MM are at risk of rejection due other HLA MM and other minor antigens.

The evidence supports LD with improved allograft survival compared with DD. Even poorly [4 to 6] MM LD have comparable outcomes with DD recipients [14]. The question is “Can a better HLA match of equal donor quality be obtained?”, whether as a 0 to 1 MM DD or through a paired exchange scheme. HLA MM is not currently the main reason for entering into paired exchange schemes [54]. However, the chances of matching would increase markedly if the numbers of potential donors increase. A chain of kidney donors has also been triggered by a DD initiating the chain and the final LD donating back into the waiting list [55]. The scenario of a poor MM LD is less common in paediatrics compared with adults (e.g. spousal donation). An online calculator based on the SRTR data is available for estimating survival times where multiple LD options are available [56]. The key to appreciating waiting times is knowing the matchability score of the patient and understanding the local kidney offering scheme. Ultimately, the decision for the type of transplant and degree of acceptable HLA MM depends on each patient circumstance and needs to take into account the added complications of ESKD and dialysis.

Early planning for transplantation is critical in paediatric practice and a ‘Transplant First’ approach remains a laudable aim [9, 57]. HLA matching is valuable for very long-term outcomes, reducing HLA sensitisation and in improving access to re-transplantation. HLA matching is crucial for reducing the ‘dialysis-free period’ over the lifetime of paediatric patients.

## Multiple-choice questions (answers following the references)

1. Which of the following is true regarding living donation (choose one)?

- Overall, living donors last longer than deceased donors.
- A living donor with 4 MM is better than a deceased donor with 1 MM of the same age.
- A living donor with haplotype MM including 1 DR MM has worse outcomes in the second transplant compared to a 0 HLA-DR MM deceased donor at the time of first transplant.
- A healthy 60 year old living donor with 3 HLA MM has worse allograft survival compared to an equivalently matched 40 year old living donor.

2. Which of the following is true regarding deceased donation (choose one)?

- There is no effect of HLA matching in young donors (<35 years of age) for 5 year allograft survival.
- Donors less than <5 years have similar survival to older donors.
- Class I HLA MM does not affect kidney transplant outcome.
- When deciding whether to accept deceased donors, one should always wait for a favourable (≤3) HLA MM.

3. Which of the following is true regarding long term allograft survival (choose one)?

- Class II HLA MM is associated with antibody mediated rejection.
- Patients who are matched for HLA-DR will always be matched for HLA-DQ.
- Median allograft survival for a 0 MM transplant is 15 years.
- 0 MM deceased donors do not reject.

4. Which of the following is true regarding long-term patient morbidity (choose one)?

- Results of registry studies can be readily applied to populations in different countries.
- MMF is a risk factor for developing cancer in transplant recipients.
- HLA-DR MM is a large risk factor for developing post-transplant lymphoproliferative disease.
- HLA MM increases risk of sensitisation after allograft loss and increases waiting times for subsequent transplants.

5. Choose the transplant with the best expected allograft survival.
  - LD from 60 year old grandmother who is a 4 HLA MM
  - LD from 30 year old father who is 3 HLA MM
  - DD from a 30 year old 3 HLA MM who died from a road traffic accident
  - DD from a 50 year old 1 HLA MM who was hypertensive and died of a cerebral vascular accident
